# 4-Methoxy­benzohydrazide

**DOI:** 10.1107/S1600536809021345

**Published:** 2009-06-10

**Authors:** Uzma Ashiq, Rifat Ara Jamal, Muhammad Nawaz Tahir, Sammer Yousuf, Islam Ullah Khan

**Affiliations:** a Department of Chemistry, University of Karachi, Karachi 75270, Pakistan; b Department of Physics, University of Sargodah, Sagodah, Pakistan; c HEJ Research Institute of Chemistry, International Center for Chemical and Biological Sciences, University of Karachi, Karachi 75270, Pakistan; d Department of Chemistry, Government College University, Lahore, Pakistan

## Abstract

The title compound, C_8_H_10_N_2_O_2_, is stabilized by three inter­molecular hydrogen bonds of the N—H⋯O and N—H⋯N types. Two intra­molecular inter­actions of the N—H⋯O and C—H⋯O types are also observed.

## Related literature

For related structures see: Ashiq, Jamal *et al.* (2008 ▶); Jamal *et al.* (2008 ▶), Kallel *et al.* (1992 ▶); Saraogi *et al.* (2002 ▶); For the biological activity of hydrazides, see: Ara *et al.* (2007 ▶); Ashiq, Ara *et al.* (2008 ▶); El-Emam *et al.* (2004 ▶); Maqsood *et al.* (2006 ▶).
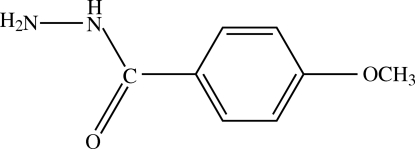


## Experimental

### 

#### Crystal data

C_8_H_10_N_2_O_2_
*M* *_r_* = 166.18
Orthorhombic, 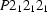

*a* = 3.9887 (1) Å
*b* = 6.1487 (2) Å
*c* = 32.8919 (9) Å
*V* = 806.68 (4) Å^3^
*Z* = 4
Mo *K*α radiation
μ = 0.10 mm^−1^
*T* = 296 K
0.22 × 0.12 × 0.10 mm

#### Data collection

Bruker Kappa APEXII CCD diffractometer
Absorption correction: multi-scan (*SADABS*; Bruker, 2005 ▶) *T*_min_ = 0.979, *T*_max_ = 0.992
17597 measured reflections
1288 independent reflections
1052 reflections with *I* > 2σ(*I*)
*R*_int_ = 0.035

#### Refinement

*R*[*F* ^2^ > 2σ(*F* ^2^)] = 0.043
*wR*(*F* ^2^) = 0.147
*S* = 1.03
1288 reflections
119 parameters
H atoms treated by a mixture of independent and constrained refinement
Δρ_max_ = 0.39 e Å^−3^
Δρ_min_ = −0.24 e Å^−3^

### 

Data collection: *APEX2* (Bruker, 2007 ▶); cell refinement: *SAINT* (Bruker, 2007 ▶); data reduction: *SAINT*; program(s) used to solve structure: *SHELXS97* (Sheldrick, 2008 ▶); program(s) used to refine structure: *SHELXL97* (Sheldrick, 2008 ▶); molecular graphics: *ORTEP-3 for Windows* (Farrugia, 1997 ▶); software used to prepare material for publication: *SHELXL97*.

## Supplementary Material

Crystal structure: contains datablocks I, global. DOI: 10.1107/S1600536809021345/pv2163sup1.cif

Structure factors: contains datablocks I. DOI: 10.1107/S1600536809021345/pv2163Isup2.hkl

Additional supplementary materials:  crystallographic information; 3D view; checkCIF report

## Figures and Tables

**Table 1:** Hydrogen-bond geometry (Å, °)

*D*—H⋯*A*	*D*—H	H⋯*A*	*D*⋯*A*	*D*—H⋯*A*
N1—H1⋯N2^i^	0.83 (4)	2.16 (4)	2.961 (3)	162 (3)
N2—H2*A*⋯O1	0.92 (5)	2.42 (4)	2.729 (2)	100 (3)
N2—H2*A*⋯O1^ii^	0.92 (5)	2.44 (4)	3.026 (2)	122 (3)
N2—H2*B*⋯O1^iii^	0.93 (4)	2.07 (4)	2.991 (2)	170 (4)
C7—H7⋯O1	0.93	2.47	2.781 (3)	100

Symmetry codes: (i) 
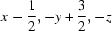
; (ii) 
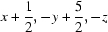
; (iii) 
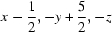
.

**Fig. 1.**
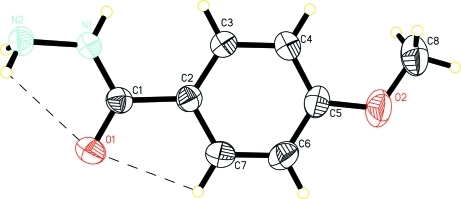
The molecular structure of (I) with displacement ellipsoids drawn at 50% probability level. The dashed lines indicates the intramolecular interactions.

**Fig. 2.**
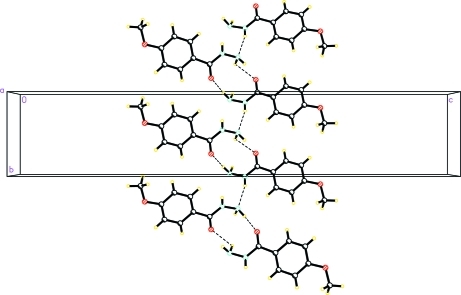
A packing diagram of (I). Hydrogen bonds are shown by dashed lines.

## supplementary crystallographic information

### Comment

Hydrazides are known to have different biological activities and have been used for the synthesis of various heterocyclic compounds (El-Emam *et al.*, 2004). The title compound was found to be antileishmanial, antibacterial and antifungal (Maqsood *et al.*, 2006). Vanadium complex of the title compound was found to ba a good inhibitor of urease (Ara *et al.*, 2007) and alpha-glucosidase (Ashiq, Ara *et al.*, 2008). In order to study the biological behaviour of 4-methoxybenzhydrazide and to investigate the change in activity due to complexation with vanadium center, we have synthesized (I) and report its crystal structure in this paper. The structures of benzhydrazide (Kallel *et al.*, 1992), *para*-chloro (Saraogi *et al.*, 2002), *para*-bromo (Ashiq, Jamal *et al.*, 2008) and *para*-iodo (Jamal *et al.*, 2008) analogues of (I) have already been reported.

The crystal structure of the title compound is presented in Fig. 1. The bond distances and bond angles in (I) are similar to the corresponding distances and angles reported in the structures quoted above. The phenyl group (C2—C7) and hydrazide moiety, O1/N1/N2/C1, in (I) are each planar with a dihedral angle between their least square planes being 7.08 (14)%. In the crystal structure, the molecules of I are linked by the N1—H1···N2, N2—H2A···O1 and N2—H2B···O1 intermolecular hydrogen bonds to form chains (details are given in Table 1, Fig 2). The geometry of 4-methoxybenzhydrazide is stabilized by N2—H2A···O1 and C7—H7···O1 intramolecular hydrogen interactions.

### Experimental

All reagent-grade chemicals were obtained from Aldrich and Sigma Chemical companies and were used without further purification. To a solution of ethyl-4-methoxybenzoate (3.6 g, 20 mmol) in 75 ml ethanol, hydrazine hydrate (5.0 ml, 100 mmol) was added. The mixture was refluxed for 5 h and a solid was obtained upon removal of the solvent by rotary evaporation. The resulting solid was washed with hexane to afford 4-methoxybenzhydrazide (yield 64%). (Maqsood *et al.*, 2006).

### Refinement

An absolute structure was not established due to lack of sufficient anomalous dispersion effects. Therefore, Friedel pairs (236) were merged. H atoms were positioned geometrically, with C—H = 0.93 and 0.96 Å for aromatic and methyl C-atoms and constrained to ride on their parent atoms. The H-atoms attached to N1 and N2 atoms were taken from Fourier synthesis and their coordinates were refined. The thermal parameter of H-atoms were: U_iso_(H) = 1.5U_eq_(methyl C) and 1.2U_eq_(the rest of the parent atoms).

### Crystal data

**Table:**

C_8_H_10_N_2_O_2_	*F*(000) = 352
*M**_r_* = 166.18	*D*_x_ = 1.368 Mg m^−^^3^
Orthorhombic, *P*2_1_2_1_2_1_	Mo *K*α radiation, λ = 0.71073 Å
Hall symbol: P 2ac 2ab	Cell parameters from 1288 reflections
*a* = 3.9887 (1) Å	θ = 1.2–28.7°
*b* = 6.1487 (2) Å	µ = 0.10 mm^−^^1^
*c* = 32.8919 (9) Å	*T* = 296 K
*V* = 806.68 (4) Å^3^	Needle, colourless
*Z* = 4	0.22 × 0.12 × 0.10 mm

### Data collection

**Table:**

Bruker Kappa APEXII CCD diffractometer	1288 independent reflections
Radiation source: fine-focus sealed tube	1052 reflections with *I* > 2σ(*I*)
graphite	*R*_int_ = 0.035
Detector resolution: 7.40 pixels mm^-1^	θ_max_ = 28.7°, θ_min_ = 1.2°
ω scans	*h* = −5→5
Absorption correction: multi-scan (*SADABS*; Bruker, 2005)	*k* = −8→8
*T*_min_ = 0.979, *T*_max_ = 0.992	*l* = −44→44
17597 measured reflections	

### Refinement

**Table:**

Refinement on *F*^2^	Secondary atom site location: difference Fourier map
Least-squares matrix: full	Hydrogen site location: inferred from neighbouring sites
*R*[*F*^2^ > 2σ(*F*^2^)] = 0.043	H atoms treated by a mixture of independent and constrained refinement
*wR*(*F*^2^) = 0.147	*w* = 1/[σ^2^(*F*_o_^2^) + (0.1097*P*)^2^ + 0.027*P*] where *P* = (*F*_o_^2^ + 2*F*_c_^2^)/3
*S* = 1.03	(Δ/σ)_max_ < 0.001
1288 reflections	Δρ_max_ = 0.39 e Å^−^^3^
119 parameters	Δρ_min_ = −0.24 e Å^−^^3^
0 restraints	Extinction correction: *SHELXL97* (Sheldrick, 2008), Fc^*^=kFc[1+0.001xFc^2^λ^3^/sin(2θ)]^-1/4^
Primary atom site location: structure-invariant direct methods	Extinction coefficient: 0.058 (15)

### Special details

**Table:**

Geometry. Bond distances, angles *etc*. have been calculated using the rounded fractional coordinates. All su's are estimated from the variances of the (full) variance-covariance matrix. The cell e.s.d.'s are taken into account in the estimation of distances, angles and torsion angles
Refinement. Refinement of *F*^2^ against ALL reflections. The weighted *R*-factor *wR* and goodness of fit *S* are based on *F*^2^, conventional *R*-factors *R* are based on *F*, with *F* set to zero for negative *F*^2^. The threshold expression of *F*^2^ > σ(*F*^2^) is used only for calculating *R*-factors(gt) *etc*. and is not relevant to the choice of reflections for refinement. *R*-factors based on *F*^2^ are statistically about twice as large as those based on *F*, and *R*- factors based on ALL data will be even larger.

### Fractional atomic coordinates and isotropic or equivalent isotropic displacement parameters (Å^2^)

**Table:**

	*x*	*y*	*z*	*U*_iso_*/*U*_eq_	
O1	0.9709 (4)	1.2503 (3)	0.05610 (4)	0.0459 (5)	
O2	0.2810 (5)	0.8530 (3)	0.21180 (5)	0.0600 (6)	
N1	0.8024 (5)	0.9374 (3)	0.02782 (5)	0.0384 (5)	
N2	0.9644 (5)	0.9802 (3)	−0.00972 (5)	0.0376 (5)	
C1	0.8272 (5)	1.0744 (3)	0.05921 (5)	0.0320 (5)	
C2	0.6754 (5)	1.0039 (3)	0.09850 (5)	0.0325 (5)	
C3	0.5396 (7)	0.8001 (4)	0.10519 (6)	0.0427 (6)	
C4	0.4053 (6)	0.7433 (4)	0.14251 (6)	0.0436 (7)	
C5	0.4061 (6)	0.8925 (4)	0.17369 (6)	0.0415 (6)	
C6	0.5410 (8)	1.0960 (4)	0.16743 (6)	0.0505 (8)	
C7	0.6748 (6)	1.1507 (4)	0.13034 (7)	0.0441 (7)	
C8	0.1357 (9)	0.6492 (5)	0.21996 (8)	0.0627 (10)	
H1	0.694 (10)	0.822 (6)	0.0282 (9)	0.0752*	
H2A	1.157 (12)	1.055 (6)	−0.0037 (11)	0.0940*	
H2B	0.832 (12)	1.068 (6)	−0.0262 (10)	0.0940*	
H3	0.53858	0.69896	0.08416	0.0513*	
H4	0.31513	0.60542	0.14648	0.0523*	
H6	0.54148	1.19702	0.18846	0.0606*	
H7	0.76633	1.28832	0.12659	0.0529*	
H8A	0.05128	0.64745	0.24729	0.0940*	
H8B	0.30181	0.53746	0.21686	0.0940*	
H8C	−0.04508	0.62368	0.20129	0.0940*	

### Atomic displacement parameters (Å^2^)

**Table:**

	*U*^11^	*U*^22^	*U*^33^	*U*^12^	*U*^13^	*U*^23^
O1	0.0595 (10)	0.0336 (8)	0.0445 (8)	−0.0109 (8)	0.0000 (7)	0.0023 (7)
O2	0.0770 (12)	0.0652 (12)	0.0379 (9)	0.0043 (11)	0.0195 (8)	0.0041 (8)
N1	0.0521 (10)	0.0313 (9)	0.0317 (8)	−0.0065 (9)	0.0048 (7)	0.0016 (7)
N2	0.0451 (9)	0.0348 (10)	0.0329 (8)	0.0030 (8)	0.0062 (7)	0.0040 (7)
C1	0.0350 (9)	0.0285 (9)	0.0325 (9)	0.0020 (8)	−0.0038 (7)	0.0031 (8)
C2	0.0352 (9)	0.0317 (9)	0.0306 (9)	0.0028 (8)	−0.0027 (7)	0.0018 (8)
C3	0.0593 (12)	0.0363 (11)	0.0326 (9)	−0.0064 (11)	0.0021 (10)	−0.0029 (8)
C4	0.0544 (12)	0.0384 (12)	0.0379 (10)	−0.0068 (11)	0.0031 (9)	0.0044 (9)
C5	0.0457 (10)	0.0469 (13)	0.0318 (10)	0.0076 (10)	0.0033 (8)	0.0031 (9)
C6	0.0708 (16)	0.0418 (13)	0.0390 (11)	0.0024 (13)	0.0038 (11)	−0.0096 (10)
C7	0.0566 (12)	0.0318 (11)	0.0438 (11)	−0.0025 (11)	0.0022 (11)	−0.0023 (9)
C8	0.0665 (16)	0.0722 (19)	0.0493 (14)	0.0039 (17)	0.0159 (12)	0.0163 (13)

### Geometric parameters (Å, °)

**Table:**

O1—C1	1.228 (3)	C3—C4	1.384 (3)
O2—C5	1.371 (3)	C4—C5	1.376 (3)
O2—C8	1.407 (4)	C5—C6	1.378 (4)
N1—N2	1.418 (2)	C6—C7	1.373 (3)
N1—C1	1.336 (2)	C3—H3	0.9300
N1—H1	0.83 (4)	C4—H4	0.9300
N2—H2A	0.92 (5)	C6—H6	0.9300
N2—H2B	0.93 (4)	C7—H7	0.9300
C1—C2	1.492 (2)	C8—H8A	0.9600
C2—C7	1.383 (3)	C8—H8B	0.9600
C2—C3	1.383 (3)	C8—H8C	0.9600
			
C5—O2—C8	118.84 (19)	O2—C5—C6	116.1 (2)
N2—N1—C1	121.47 (17)	C5—C6—C7	120.5 (2)
C1—N1—H1	124 (2)	C2—C7—C6	120.9 (2)
N2—N1—H1	114 (2)	C2—C3—H3	119.00
N1—N2—H2B	111 (3)	C4—C3—H3	119.00
H2A—N2—H2B	108 (4)	C3—C4—H4	120.00
N1—N2—H2A	107 (2)	C5—C4—H4	120.00
O1—C1—N1	121.69 (17)	C5—C6—H6	120.00
N1—C1—C2	117.14 (17)	C7—C6—H6	120.00
O1—C1—C2	121.17 (16)	C2—C7—H7	120.00
C1—C2—C7	117.86 (18)	C6—C7—H7	120.00
C3—C2—C7	118.06 (18)	O2—C8—H8A	109.00
C1—C2—C3	124.08 (16)	O2—C8—H8B	109.00
C2—C3—C4	121.4 (2)	O2—C8—H8C	109.00
C3—C4—C5	119.5 (2)	H8A—C8—H8B	109.00
O2—C5—C4	124.2 (2)	H8A—C8—H8C	109.00
C4—C5—C6	119.7 (2)	H8B—C8—H8C	109.00
			
C8—O2—C5—C4	−1.3 (4)	C7—C2—C3—C4	0.2 (4)
C8—O2—C5—C6	179.2 (3)	C1—C2—C7—C6	−179.6 (2)
N2—N1—C1—O1	5.3 (3)	C3—C2—C7—C6	−0.5 (4)
N2—N1—C1—C2	−174.18 (18)	C2—C3—C4—C5	0.1 (4)
O1—C1—C2—C3	−173.4 (2)	C3—C4—C5—O2	−179.7 (2)
O1—C1—C2—C7	5.7 (3)	C3—C4—C5—C6	−0.2 (4)
N1—C1—C2—C3	6.1 (3)	O2—C5—C6—C7	179.6 (2)
N1—C1—C2—C7	−174.9 (2)	C4—C5—C6—C7	−0.1 (4)
C1—C2—C3—C4	179.3 (2)	C5—C6—C7—C2	0.4 (4)

### Hydrogen-bond geometry (Å, °)

**Table:**

*D*—H···*A*	*D*—H	H···*A*	*D*···*A*	*D*—H···*A*
N1—H1···N2^i^	0.83 (4)	2.16 (4)	2.961 (3)	162 (3)
N2—H2A···O1	0.92 (5)	2.42 (4)	2.729 (2)	100 (3)
N2—H2A···O1^ii^	0.92 (5)	2.44 (4)	3.026 (2)	122 (3)
N2—H2B···O1^iii^	0.93 (4)	2.07 (4)	2.991 (2)	170 (4)
C7—H7···O1	0.93	2.47	2.781 (3)	100

Symmetry codes: (i) *x*−1/2, −*y*+3/2, −*z*; (ii) *x*+1/2, −*y*+5/2, −*z*; (iii) *x*−1/2, −*y*+5/2, −*z*.
